# Narratives of Italian Transatlantic (re)migration, 1897–1936

**DOI:** 10.3389/fsoc.2023.1239585

**Published:** 2023-11-22

**Authors:** Lorella Viola

**Affiliations:** Luxembourg Centre for Contemporary and Digital History, University of Luxembourg, Esch-sur-Alzette, Luxembourg

**Keywords:** remigration, Italian American migration, ethnic press, discoursehistorical analysis, digital humanities, identity

## Abstract

Remigration is typically envisioned as the final stage of the migration experience, a one-way movement from the host country to the country of origin. This article offers a novel, intimate view of historical return migration as a more complex and discursive process. The case study is Italian American migrants at the turn of the twentieth century, one of the groups which – according to historical statistics – was most actively engaged in Transatlantic remigration; more recent readings, however, show that many of these returnees eventually re-emigrated to the US. Using for the first time immigrant newspapers against the baseline of the Italian public discourse, the article analyzes Italian migrants’ own accounts of remigration as a way to access the more subjective dimension of migration. The integration of text mining and Critical Discourse Analysis will show that migrants were experiencing migration as a sense of identity crisis manifested through feelings of being misunderstood, rejected and unappreciated. These results indicate a less material reading of (re)migration, that is beyond economic reasons, and that for many individuals remigration was a bi-directional movement, only fully concluded when they were no longer experiencing a sense of identity crisis, be it in their homeland or the host society. The article will argue that this was the visible outward sign of a much more profound issue: the Italian Government’s view of (r)emigration –mainly through the lens of domestic economic advantage –deeply underestimated the complexity of migration as a social phenomenon and as a profoundly changing psychological experience. In the long run, this error of judgment deeply damaged Italy as many of those *ritornati* felt misunderstood and disillusioned and crossed the Atlantic again, this time never to return.

## Introduction

Since the early mass migration movements of the past centuries, return migration has always represented a rather complex and critical aspect. Although generally less researched than other topics, studies on the subject has on the one hand primarily revolved around the *impact* of return migration, that is around the one question of whether return migration is beneficial or detrimental, especially economically but also socially, particularly for the sending countries (Saloutos, 1956; Boyd Betty, 1973; Bohning, 1975; Böhning, 1975; Rosoli, 1977; Musillo, 1981; Gentileschi and Simoncelli, 1983; Wyman, 1993; Bonifazi and Heins, 1996). On the other, research has investigated the reasons for returning, positioning the discussion along the conceptual binary of integration/failure to integrate in the receiving country (Cerase, 1967, 1974; Rogers, 1983; Kubát and Center for Migration Studies, 1984). Within the first orientation, a pioneering book is *They remembered America* Saloutos (1956) which investigates the impact of repatriated Greek Americans onto Greek society. From the analysis of two-hundred interviews, the author explores both the reasons behind the repatriation and the potential larger significance for Greek society. Although it may be difficult to generalize conclusions due to the small sample and the highly qualitative focus, the results suggest a minimal overall impact of the returnees on the sending country. This would be explained by returnees’ ambivalent sentiments towards their return experience as well as no ability or desire to Americanize Greece.

Another study which assesses the socio-economic impact of the migratory flows particularly for the sending countries is that by Bohning (1975). Geared more towards exploring issues of development and employment, Bohning’s analysis of return migration focuses on emigration statistics from the Mediterranean mostly to western European countries. The book questions the supposed benefits of return migration brought by emigration specifically, and argues against a “*laissez-faire*” policy. Focussing on the Italian case, Rosoli’s analysis (Rosoli, 1977) of return migration reaches the same conclusions as Bohning’s (op. cit.), but it also highlights the importance of addressing the complexity of the migratory phenomenon not solely from a political and economic perspective, but also crucially in terms of the collective experience understood as the sum of migrant’s sacrifices and challenge’s once back (244).

Musillo (1981) survey also attempts to answer some of the questions concerning the benefits of migrants’ return for their country of origin, but it does so through the analysis of employment rates and interviews of a small (200) sample of Italian migrants who returned from Switzerland to southern Italy between 1969 and 1970. Specifically, this work aims to investigate the causes of return migration in relation to the reception policies implemented by the Italian national and regional authorities. The results again suggest a significant discrepancy between return policies and any concrete benefit.

In relation to the investigation of the reasons for returning, Rogers (1983) analyzes census and survey data from several countries and cross-examines them with findings from previous studies. Her results highlight some of the gaps in research on return migration at the time, including the lack of cultural indicators in the surveys, sample limitedness, and the inadequacy of a persistent model that sees the migrant who returns essentially as a failure to integrate into the host society. Though still in relation to the time when the study was carried out, the conclusions point to the need for a more nuanced investigation of the phenomenon of return migration that would go beyond the binary conceptual framework of whether it is good or bad.

Gentileschi and Simoncelli (1983) address the specific question if and to what extent return migration rebalanced regional and local differences. From the review of relevant data for Italy of the previous twenty years, the book concludes that return migration has not had any significant effect on the territorial imbalance. The authors also suggest that research on the topic should consider the migrant’s family rather than the individual, thus opening interesting avenues for a more cognitive and cultural angle. Kubát and Center for Migration Studies (1984) analyze return migration in Europe from a socio-political perspective. The book attempts to map the impact of return migration on the sending countries in relation to restrictive measures in the receiving countries as well as integration policies in the sending countries aimed at encouraging return. From data on Algeria, Tunisia, Spain, Portugal, Italy, Greece, former Yugoslavia, France and Turkey among others, the book concludes that “emigration has failed to provide a discernible developmental impetus in any of the countries” (*ibid.*, 266). More recently, Wyman (1993) draws on official U.S. and European statistics on returned migrants, previous studies on the experience of return migrants, and his own archival work to shed light on the migrants’ motivations to return. The author highlights commonalities and differences between national groups such as the fact that for many Europeans, the intention had never been to settle in America, and so returning was not the result of a failure, but an undisputed decision.

As this brief literature review shows, these important contributions all acknowledge the relevance of integrating the study of return migration with a more comprehensive analysis of the cognitive and subjective aspects of the migratory experience. Methodologically however, these works reflect a general tendency to either take a macro approach based on the analysis of historical events, statistics and socio-economic indicators or to adopt a zoom-in perspective based on a limited amount of small case studies. There seems to be a frustrating lack of large-scale investigations that would devote attention to the cultural perception of return migration –both by the country of origin and by migrants themselves –as an alternative entry point to the question of *why* they returned. In other words, historical analyzes that focus on the inner voice of migrants and on *how* they perceived returning beyond the reasons for returning remain comparatively rare. The reasons for that are complex but on the whole scarcity of primary sources may be at the root of the gap. Moreover, for the most part remigration is rigidly envisioned as a one-way movement from the host country to the country of origin and, perhaps even more critically, as the final stage of the migration experience.

This article explores a novel way to understand historical return migration as a more complex and discursive process. It does so by using migrants’ own narratives of migration both as a data collection tool and an analytical object (Baynham and De Fina, 2005; De Fina, 2017; Viola, 2021). Without losing the quantitative advantage of large-scale data, the aim is to obtain a more intimate investigation of return migration than statistics, analysis of public discourse alone or small, post-facto interviews can offer that could allow us to explore unanswered questions about the mass migratory movements of the past century. The case study is Italian American migrants at the turn of the twentieth century. The study uses as its main primary source *ChroniclItaly 3.0* (Viola and Fiscarelli, 2021), a collection of Italian diasporic newspapers published in the United States between 1897 and 1936. By using diasporic newspapers –newspapers written by migrants for the migrants –the analysis explores migrants’ stories of their own migratory experience (Viola, 2021).

The study rests on two hypotheses. The first one is that the Italian domestic discourse about remigration was significantly different from the Italian American one. We base our hypothesis on the fact that discourses of migration (emigration, immigration, and remigration) are always built on justification and legitimation (especially when societies feel threatened) as well as moral values and fluctuate with the development of economic needs and political agendas (Viola and Verheul, 2020b; Oberbichler and Viola, 2023). The immigrant press, on the contrary –though certainly mediated –may offer a more authentic voice of the migratory experience in that it was produced by the diasporic community itself. To test this hypothesis, the analysis will compare the narratives constructed in the Italian American newspapers and used by migrants to communicate (return) migration with the baseline of public discourse of emigration and remigration in Italy.

The second hypothesis is that remigration is a movement both from the host country to the country of origin and vice versa. Historical scholarship of migration has widely acknowledged the phenomenon of the so-called “birds of passage,” that is temporary migration of mostly young males who traveled back and forth depending on the accumulated capital when abroad. This pattern is shared by many, if not all national groups, including of course Italy. This means that the final decision of settling in America or returning to the home country was often the result of several transatlantic trips, sometimes in the span of years during which these individuals, whilst living through larger historical changes, learned from the experience and were profoundly changed by it. Within this framework, the displacement process of remigration, it is argued here, can equally be conceptualized as a crisis of identity, in both collective and personal identities. This is justified by the fact that social processes of displacement and transformation deeply affect people’s inner notions of identity and belonging. For example, in the context of Italian Transatlantic migration, historical statistics show that Italians were one of the groups most actively engaged in Transatlantic remigration but more recent readings indicate that many of these returnees eventually re-emigrated to the US (Boyd Betty, 1973). Indeed, one of the open questions about (r)emigration is the discrepancy between migrants’ statements at the departing ports and their actual behaviors. By exploring this more subjective element, this study may expand on the current conceptualization of return migration as the ending point of the experience.

For example, according to Cerase (1967), there are four different paradigms of return migration: failure, conservation, investment, and retirement. The first one would describe a failure of the migration project, for instance a failure of integration in the host country, and it would entail a rapid re-integration in the country of origin. The second one, the migrant of conservation would be the one who has kept ties with the country of origin, for instance through several trips and who, once accumulated enough capital, perhaps after five to ten to years, decides to return to the society of origin, but we no ability to impact it in any way. The migrant of investment would describe a type of migrant who has internalized new values in the host country and once achieved their personal goals, is eager to contribute them to the old society. Finally, the migrant of retirement would be the type of migrant who only returns once the accumulated capital allows them to live comfortably in the country of origin without having to work and who, consequently, would not be interested in contributing to the society of return. Cerase’s model may therefore explain whether remigration is beneficial or detrimental for the sending country in relation to the reasons for returning. However, even though it recognizes the ‘birds of passage’ pattern, it rigidly understands return migration as the final stage of the migration experience. In other words, it fails to acknowledge that the experience often did not end with repatriation. Indeed, returnees did not always meet favorable circumstances on their return and in the mid-, long term, the difficult readjustment disappointed their expectations. The skills supposedly acquired abroad very rarely fitted the economies of the places of origin and any impulse of innovation often found major socio-cultural and political obstacles (Rosoli, 1977). It is therefore not unreasonable to hypothesize that particularly for the migrant of investment –the only one truly capable of representing an innovative contribution to the society of origin –remigration abroad became the only alternative. This study wants to explore this more complicated pattern of migration. Resting on the foundation that discourses on migration also inevitably go along with a sense of crisis (Viola and Musolff, 2019, 3), we posit that the more nuanced experience of migration provided by immigrants’ newspapers will narrate the intricate relationships between specific manifestations and negotiations of identity and the wider experience of migration as embedded in the larger social context of both the American and the Italian society of the time.

Another distinctive feature of this study is the integration of Critical Discourse Analysis theory (Fairclough and Wodak, 1997) into the quantitative investigations (collocations and ngrams). *ChroniclItaly 3.0*, the archive used for the investigations, contains 21,454,455 words, therefore using close reading methods alone would not allow us to make full use of the record. The proposed methodology will first provide a comprehensive, zoom-out overview of how words are distributed in the newspapers and second, will identify relevant narratives. CDA will then be used as the applied theory for the analysis of the narratives of (r)emigration. Despite the ever-large amount of available digital sources, particularly for historical research, to my knowledge only two studies exist to date that have conducted digital discourse-based analysis of remigration on large quantities of textual data (Oberbichler and Pfanzelter, 2022; Oberbichler and Viola, 2023). The methodology proposed in this research may therefore have wider relevance for historians and other humanities researchers who are increasingly confronted with the challenge of having to navigate the complexity of sources abundance, particularly historical.

Finally, as an additional innovative contribution, this study will be the first to use immigrant newspapers in remigration scholarship to explore the tension between questions of policy and governance as manifested through language in the public discourse and the individual’s actual experience as it emerges from their own discourse of migration. Though certainly considered as an important source for migration history, immigrant newspapers have comparatively received less attention than national media, especially in digital research, because they were believed to be considerably less worthy of scrutiny. To the contrary, as recent digital research on migration and identity has shown (Viola and Verheul, 2019a,b, 2020a; Viola, 2021) and as it will be further demonstrated here, immigrant newspapers offer unique emic perspectives on how migration was perceived by migrants through how it was narrated to migrants themselves. In this way, this study also actively engages in the debate about language diversity representation and archival biases in digital practices.

### Italian Transatlantic (r)emigration

For the period of mass migrations (1860–1920), official statistics indicate that about 40% of all European migrants eventually returned. More recent studies, however, evidence that due to differences in how the data were gathered between sending and receiving countries and several errors in how individuals were counted, the return migration rate from the host country to the country of origin may have in fact been as high as 70% (Gould, 1980; Bandiera et al., 2013; Dustmann and Görlach, 2016; Abramitzky et al., 2019). As for Italy, the most accurate data on return migration were gathered by the *Commissariato Generale dell’ Emigrazione* (general commissariat for emigration) in the years 1905–1906 (Beneduce, 1910). The survey also compared rates of return in 1905–6 with rates of emigration in 1901–1905, this is because available statistics at the time indicated that most Italians were staying in America between two to five years. According to these data, *I Ritornati* (the returnees), as they were called, were about 40%, they were predominantly males (90%), young (between 14 and 44), uneducated and unskilled. Women were less likely to return because they would typically emigrate to join their husbands who had already decided to settle abroad. Although certainly valuable, these data suffer from severe limitations; above all, they exclude from the count the returnees entering Italy through ports other than Genoa, Naples, Palermo, and Messina, or by railroads.

In the United States, data on return migration were gathered from 1907 to 1908 by the Commissioner of Immigration. As noted by Gould (1980), however, migration statistics based on these numbers give an inaccurate picture of the actual intensity of return migration, or migration in general for that matter, because the data collection was based on the statement of the individual about their intention to stay abroad or to return –not actual figures. Latin American surveys are even less reliable. Nevertheless, both Italian and American statistics relatively consistently show that although Italians were the largest community of immigrants, they were also the group most actively engaged in return migration. There seems to be consensus now in the literature that between 1900 and 1920, at least 50% of individuals returned, though again this percentage varies greatly according to the year and the region in Italy (Boyd Betty, 1973).

Another percentage that is of particular interest is the one indicating the number of individuals the Dillingham Commission^1^ referred to as *non-emigrant aliens*, that is non-US individuals who had declared that they would return to the United States within a year. These figures show a steady decreasing trend therefore suggesting that most returnees intended to resettle permanently in Italy (United States Bureau of Immigration and Naturalization, 1908). However, crossing data from Italian departures and American arrival statistics, the numbers tell a different story. They show that even if most returnees had initially intended to resettle permanently in Italy, they eventually re-emigrated to the US as indeed, second or even third departures were a common phenomenon happening at intervals of five to ten years (Cerase, 1967). By investigating Italian migrants’ own narratives of migration, this study explores the potential reasons for re-emigrating to the US.

### The Italian debate of (r)emigration at the turn of twentieth century

The political unification of Italy (1861) had dramatically worsened the already critical economic situation of the South, making the task of creating a nationally integrated economy virtually impossible (Viola, 2019). The rapid impoverishment of the South, the *Questione meridionale* (the southern question) as it was called, soon became the government’s top priority, therefore dominating the public debate (Wong, 2006). However, despite several targeted programs and special legislations geared towards solving it, three decades later the delay of the South was worse than ever.

It is in these years that discourses on migration (both emigration and return migration) entered the public debate. Emigration was generally considered as a negative phenomenon, seen as a disruption of the social and moral order, a drain of capital, and a side-effect of the process of national integration which ultimately made Italy look bad abroad (di Cosentino, 1873). Accordingly, the Italian Government was against emigration (Manzotti, 1962; Ostuni, 2001; Choate, 2008). However, despite efforts to limit emigration, towards the end of the century migrating movements, especially from the South to Europe and America, reached numbers so impressive that the Government had to change its strategy. And because the highest emigration flows were departing from the South, naturally the topic of emigration became entangled with the *Questione meridionale* (Manzotti, 1962) and more precisely, framed as a way to solve it. After decades of failed attempts at rescuing the South, politicians started to argue that there was nothing that the Government could do to help the cause because, in fact, the South could not be helped (Wong, 2006). Concurrently, some economists began to highlight the positives of emigration. Emigration was relieving the South of Italy –and therefore the country as a whole –from the demographic burden of unemployment. Moreover, thanks to migrants’ remittances, Italy was experiencing an unprecedented flow of cash which in the long term would have made the South –and therefore the country as a whole –richer. Perhaps emigration was not as disrupting as observers had originally argued only a few years before. Perhaps emigration and remittances were in fact the only way the southern question could finally be solved.

It is important to state that at the turn of the twentieth century, within the national debate of emigration Italian migrants were expected to return. This expectation was in line with the general European trend for which migrants were indeed returning. The argument was that for as long as emigration was necessary, Italians would continue to leave but these migrants would alternate on a rotating basis every three to 5 years. In other words, virtually every migrant would have eventually come back (Bertani, 1886). Moreover, so the argument went, these migrants would have returned with money and new acquired skills and their enriching experience would have contributed to the cultural modernization of the Italian society. Naturally, migrants who were not conforming were victim of harsh criticisms; if they decided to settle permanently abroad, they were framed primarily as amoral individuals or disloyal to the nation and its values, and as a failure or as parasites if they did not promptly invest their savings once back in Italy (the so called “returnees of retirement”) (Jacini, 1885; Cerase, 1967, 1974).

As the debate was shifting more towards emigration as being beneficial for Italy, the role of the Government became at the centre of the discourse. One of the main arguments was that the Government had the obligation to regulate emigration so that its benefits could be maximized, and its disrupting effects contained. In other words, regulation of emigration was in the national interest (Carpi, 1871, 1878). The discussion mostly focused on remittances and their intelligent exploitation (Balletta, 1968). Indeed, remittances were considered a true national asset, crucial to the Italian economic integration in the international capitalistic system. At the individual level, remittances may have been small, but in the aggregate, they represented substantial amounts of money. For this reason, the Government had to protect remittances every step of the journey from the rest of the world to Italy. Better yet, it had to regulate the way remittances were channeled so that immigrants’ savings could be used productively (Gatto, 2021). As the late Prime Minister Francesco Crispi claimed, after all national and individual immigrants’ goals were the same and assisted emigration was the key to reach them both (Crispi, 1915).

These arguments culminated in the 1901 law, the first Italian law on emigration. The law aimed to assist migrants in all matters concerning the migratory experience, including issuing the necessary documents, setting ships’ sanitary standards, creating guidelines for transatlantic fares, and establishing employment offices in the major American destinations. From that moment on, all these activities were to be managed by the newly established *Commissariato Generale dell’ Emigrazione*. Remittances would also start to be regulated. With this law, the Government officially gave the Banco di Napoli the task to collect, protect, save, and transfer migrants’ remittances from all over the world to Italy (Gatto, 2021). The aim was to minimize the number of intermediate actors in the transferring process, to keep commissions and exchange fees low and guarantee that the highest possible amount of money would reach Italy. The centralization of the remittances would have then protected migrants’ savings, and thus the country’s interests. However, due to both structural and cultural barriers, the plan to centralize the channeling of remittances never reached the Government’s unrealistic expectations, as most migrants would still send their money through *banchisti*,^2^ friends, relatives, or post offices. It has been calculated that in the end, only one-fourth of the remittances reached Italy through the Banco di Napoli (Commissariato Generale dell’Emigrazione, 1925).

The 1901 law remained practically the same until fascism when the regime firmly rejected emigration as weak and unpatriotic (Gentile, 1986; Choate, 2008; Braun-Strumfels et al., 2023). Fascism opposed the narrative that Italians were forced to emigrate, and it replaced the word *emigrante* (emigrant) with the more pleasant title *lavoratore italiano all’estero* (Italian worker abroad). Along with the same narrative, in 1927 the *Commissariato Generale dell’ Emigrazione* was abolished. But for more than three decades prior to fascism, the national discourse had praised migration, return migration, and remittances for single-handedly being able to achieve what decades of governmental measures had failed to accomplish. The reason behind such a naïve and opportunistic vision of (r)emigration and the hype about remittances as a miracle cure may be found precisely in the failure of all previous governmental programs. Manzotti (1962) for instance argues that it was with the Second World War that the deeper fractures in the Italian economy became ever more apparent, demonstrating that migration was only one aspect of a much wider economic crisis.

## Sources and methodology

### The immigrant press to access narratives of migration

One of this study’s most distinctive features is the use of immigrant newspapers (*ChroniclItaly 3.0-*Viola and Fiscarelli, 2021) to explore questions of belonging and identity in relation to the experience of migration and return migration. Beyond the binary conceptual framework about the function of the immigrant press in the USA, i.e., either assimilating or retarding assimilation (Hickerson and Gustafson, 2016), in the absence of large-scale accounts of migrants’ personal experience, this study explores immigrant newspapers as a way to *unsilence* the voice of migrants.

The immigrant press constitutes the first historical stage of the ethnic press and it is a phenomenon associated with the mass migration from Europe to the Americas between the 1880s and 1920s (Viola and Verheul, 2019a). During this period, it is estimated that in the USA alone ∼1,300 foreign language newspapers were read by about 2.6 million people (Rhodes, 2010; Bjork, 2013). As for the Italian immigrant press, recent calculations estimate that in the period of reference, between 150 and 264 Italian language newspapers were published in the USA, of which 98 managed to publish uninterruptedly (Deschamps, 2011, 81; Vellon, 2017). The newspapers of the years 1880–1920 are generally divided into two main categories: *prominenti* and *sovversivi.*^3^ The *prominenti* were mainstream newspapers whereas the *sovversivi* were radical publications of socialist and anarchic orientation.^4^ In terms of reach, their circulation ranged from few hundreds to many thousands (Vecoli, 1998). In his 1922 investigation of the role of the immigrant press in the USA, urban sociologist Robert E. Park reported that in 1900, 691,353 Italian newspapers were sold across the United States (Park, 1922, 304) and in New York alone, the circulation ratio of the Italian daily press was one paper for every 3.3 Italian New Yorkers (Vellon, 2017, p. 10). These already impressive numbers should however be doubled or even tripled, since most Italians were illiterate at the time and newspapers were often read aloud (Viola and Verheul, 2019b).

Despite the high distribution and circulation figures, some scholars have argued that the influence the Italian language press exerted on the immigrant community was rather limited (Russo, 1972; Vecoli, 1998, 2006). The reason for that would lie in the fact that immigrant newspapers could not impose their definition of social reality because ultimately, migrants had to make sense of it within the context of their own migratory experience. Others have stated that because they were often pushing specific individual agendas, these media in fact damaged Italians (Pozzetta, 1973). Though true to an extent, it is undisputable that by functioning as tools of language retention and national identity construction and preservation (Vellon, 2017; Viola and Verheul, 2019a,b), Italian newspapers became a powerful instrument for community building (Park, 1922; Vellon, 2017; Viola and Verheul, 2019a,b; Deschamps, 2020). Moreover, beyond individual agendas, Italian immigrant newspapers would play an important social role for example by offering practical and cultural information whilst at the same time reporting news from the homeland. Finally, they championed for the rights of the Italian immigrant community by supporting nationalistic campaigns, entering pleas for convicted Italians, holding fundraisings for natural disasters in Italy, and voicing protests against mistreatments of Italians (Vecoli, 1998).

### ChroniclItaly 3.0

The collection *ChroniclItaly 3.0* used in this study features ten titles of *prominenti*, *sovversivi*, and independent newspapers published between 1897 and 1936. *ChroniclItaly 3.0*^5^ is fully Open Access. The titles included in *ChroniclItaly 3.0* are: *L’Italia, Cronaca Sovversiva, Il Patriota, La Libera Parola, La Rassegna, La Ragione, L’Indipendente, La Sentinella, La Sentinella del West,* and *La Tribuna del Connecticut* for a total of 8,653 issues and 21,454,455 words.

**Figure 1:**
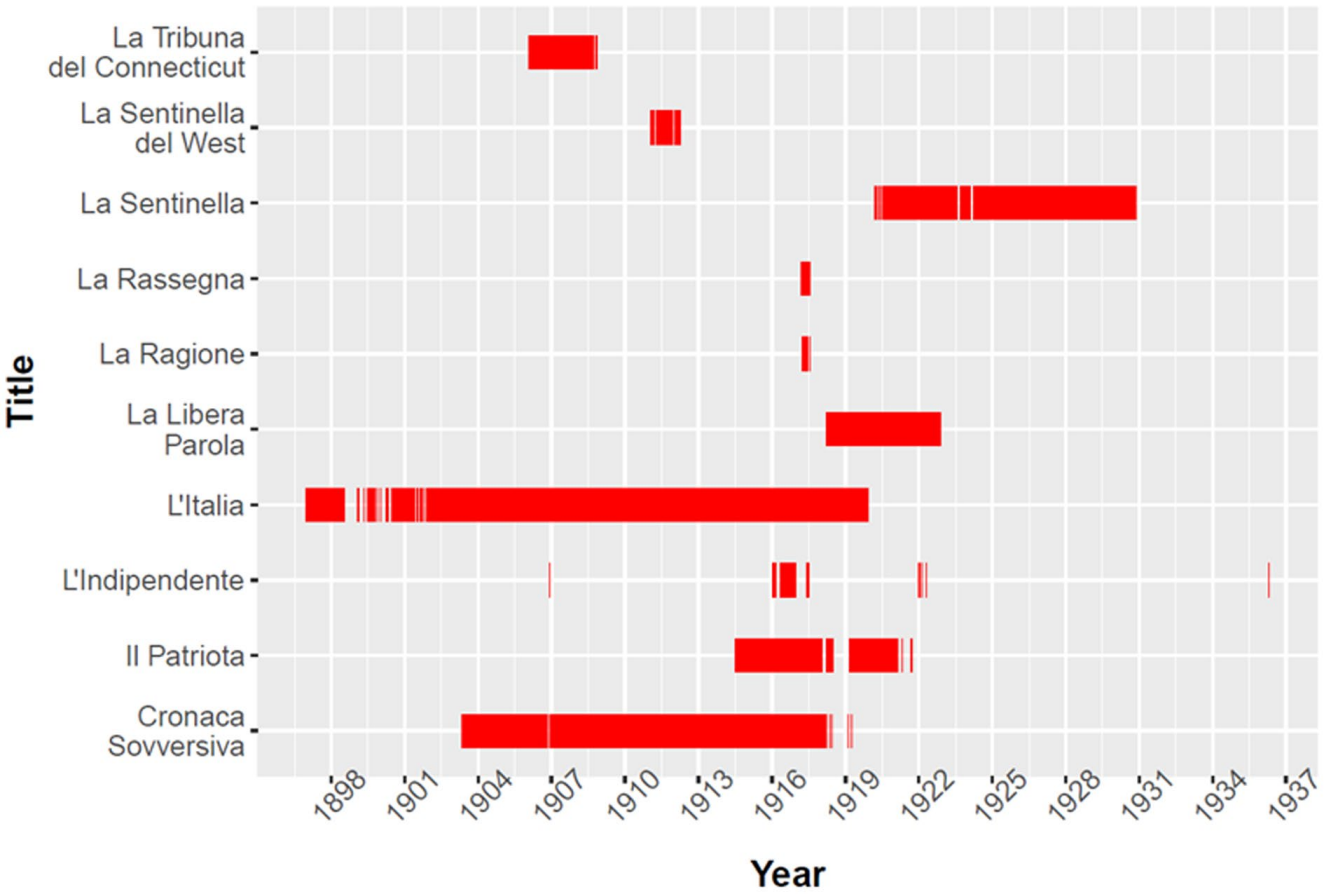
Distribution of issues within *ChroniclItaly 3.0* per title. Red lines indicate at least one issue in a three-month period.

*L’Italia* is the title with the highest number of issues in the corpus (6,489) and by far, the one that covers the longest timespan (1897–1919) (see Figure 1). The title was founded in 1886 by a group of Italian *prominenti* from the fusion of two failing papers. Initially, the newspaper was published bi-weekly, but from 1889, it was published daily. In 1895, the editor-in-chief of *L’Italia* was Pio Morbio, co-founder of *Il Corriere della Sera*, one of the main newspapers in Italy at the time, while in 1897 Ettore Patrizi and Giovanni Almagia became co-editors. In 1898, Patrizi became the sole owner and publisher of the newspaper. For about two decades, under Patrizi’s lead, *L’Italia* voiced a leftist ideology, close to the Italian labor class, and defended Italians against defamation and discrimination. However, after 1909, Patrizi overtly embraced Mussolini’s ideology and the newspaper’s orientation became ardently nationalistic.

*Cronaca sovversiva*, a *sovversivi* newspaper, is the second largest title in *ChroniclItaly 3.0*; it includes 771 issues from 1903 to 1919. It was founded by the anarchist Luigi Galleani in 1903 who had escaped extradition a few years before and had settled in Barre, Vermont, where an Italian community of stonemasons was living. Galleani published the anarchist newsletter for 15 years until the United States government forced him to stop under the Sedition Act of 1918. *Cronaca Sovversiva* typically discussed a variety of radical topics, including arguments against the existence of God and against historical and contemporary establishment. Like all radical press, *Cronaca sovversiva* not only served as the main means of communication for the *sovversivi*’s community but it acted as the movement’s financial centre (Bencivenni, 2016). Deschamps (2020) argues that within the immigrant press, it was especially the radical press that truly functioned as a transnational tool since it was published in the US but it was mainly targeted at a readership in the country of origin.

*La Sentinella* includes 518 issues from 1920 to 1930. *La Sentinella* included more pages than other Italian-language newspapers of the time, with some issues extending to a full eight pages. Interestingly, during the early 1920s, each issue included a page dedicated to news from the Italian American community of Port Chester, New York. The exact foundation date is unknown, but it might have been in 1913 or 1914.^6^ Politically, *La Sentinella* was a Republican-leaning paper and it overtly supported the Fascist ideology (Bucki, 1995).

*Il Patriota* contains 339 issues from 1914 to 1921. It was founded in 1914 in Indiana, Pennsylvania by Francesco Biamonte. It claimed to be a politically independent newspaper aiming to inform Italians in the region and to offer immigrants advice on adjusting to American life. *Il Patriota* encouraged Italians to become naturalized citizens and it permanently featured a column listing questions probably taken from the citizenship test.

*La Libera Parola* – which was originally called *La Voce del Popolo* – features 240 issues from 1918 to 1922. It was founded in 1906 in Philadelphia by the two brothers Arpino and Giovanni Di Silvestro. It was a weekly newspaper which publicized the activities of the Pennsylvania Chapter of the Order of the Sons of Italy and overall had a nationalistic orientation. For example, *La Libera Parola* supported Italy’s participation in the war and criticized Pope Benedict XV for opposing Italy’s involvement in the conflict. The paper also encouraged Italian-Americans to become American citizens, enlist in the military, and buy Liberty Bonds to help finance the Allied war effort.

*La Sentinella del West Virginia* contains 53 issues from 1911 to 1912. It was West Virginia’s only Italian periodical. It was founded by Rocco D. Benedetto in 1905 and by 1906, its circulation had peaked at 3,500 copies. Although Benedetto was an active Republican, the newspaper claimed to be politically independent. The publication mainly informed immigrants about Italy, but also about their new homeland. The paper discussed American politics and current events, but it mostly chronicled the stories of the Italian immigrants in West Virginia.

*La Tribuna del Connecticut* contains 130 issues from 1906 to 1908. It was a weekly newspaper published in Bridgeport, Connecticut where an Italian *colonia* (colony) was rapidly growing. The paper claimed to be independent and voiced support for socialism, striking laborers, and the International Workers of the World (“Wobblies,” for short). *La Tribuna del Connecticut* published reflections on America by Russian writer Maxim Gorky; gave prominent coverage to notable socialist intellectuals visiting Bridgeport, like Italian editor Carlo Tresca; and responded to articles and ideas then being discussed in Italian socialist newspapers. It also informed the Italian community about dances, concerts and other social activities. Of note is that *La Tribuna del Connecticut* also served Danbury, Connecticut and Port Chester, New York; it also had a wide network of stable regional correspondents who were located in more than a dozen cities in the Nutmeg and Empire States (Connecticut and New York). In 1913 Altieri became the editor of *La Sentinella*.^7^

*L’Indipendente* includes 48 issues from 1907 to 1936. It began publication as a weekly in 1904. It was headquartered in Wooster Square, one of two neighborhoods (along with the “Hill”) which housed New Haven’s large Italian immigrant community. It claimed to be the “first and only Italian daily in New England.” Its aim all was to support and protect Italians in New Haven. Although it claimed to be pro working classes, the tone of the coverage was pro-capitalist and pro-American which was not uncommon in the early 20th century Italian American press.^8^ Unlike other Italian American newspapers that presented it as a source of national pride, *L’Indipendente* overtly opposed the Italian colonial invasion of Ethiopia of 1936.

*ChroniclItaly 3.0* also features the whole 40 issues of *La Ragione* from 25 April to 23 August 1917, as this newspaper only survived eight editions. It was published in Philadelphia and its main aim was to expose corrupted personalities within the Italian community such as *prominenti* and dishonest bankers (*banchisti*). *La Rassegna* was also a short-lived newspaper published in Philadelphia in 1917. The archive includes 25 issues from 7 April to 25 August 1917. It focused on issues affecting Italian immigrants in Philadelphia and chronicled major historical events such as World War I and Italy’s nationalistic claims to Dalmatia. In addition to defending *prominenti*, *La Rassegna* encouraged Italian immigrants to seek naturalization.

Due to economic struggles, immigrant newspapers were often abruptly discontinued; this is reflected in the composition of the collection which presents both gaps across titles and differences in the number of issues per title as described above and shown in Figure 1. However, the factor that most heavily conditioned which titles and which issues are included in *ChroniclItaly 3.0* is the existence of a complete, or largely complete, microfilm “object of record” with priority given to higher-quality microfilms (Viola, 2023). This requirement was set by the American National Digital Newspaper Program (NDNP), the American National Endowment for the Humanities (NEH), and the Library of Congress in 2005, when the mass digitization program *Chronicling America*^9^ started. This criterion is still adopted for reasons of efficiency and cost; however, as in the past microfilming practices in the United States were entrenched in a complex web of interrelated factors (Baker, 2002), including economic and political interests, the material in the directory incorporates issues such as previous decisions of what was worth microfilming and more importantly, what was not (Rumsey and Digital Library Federation, 2001; Fagan, 2016; Viola, 2023). Though titles and issues are constantly added to the database, this element has also influenced the periodization of the collection, i.e., 1897–1936, in that it reflects the titles available in *Chronicling America* at the time when *ChroniclItaly 3.0* was harvested. In other words, *ChroniclItaly 3.0* was not created with a specific periodization in mind nor for the specific goal of studying Italian American migration. The aim was to provide a digital source of Italian American newspapers for scholars of nineteenth-century periodicals and intellectual and digital history, but more widely for historians, linguists, media and communication scholars interested in topics such as conceptual change, continuity and replacement, and representation of actors and events in public discourses. More widely still, for anybody concerned with text mining in the humanities (Viola, 2021). Nevertheless, the author acknowledges the far-reaching network of influencing factors and actors involved in digital research which have impacted the creation of *ChroniclItaly 3.0*.

**Figure 2:**
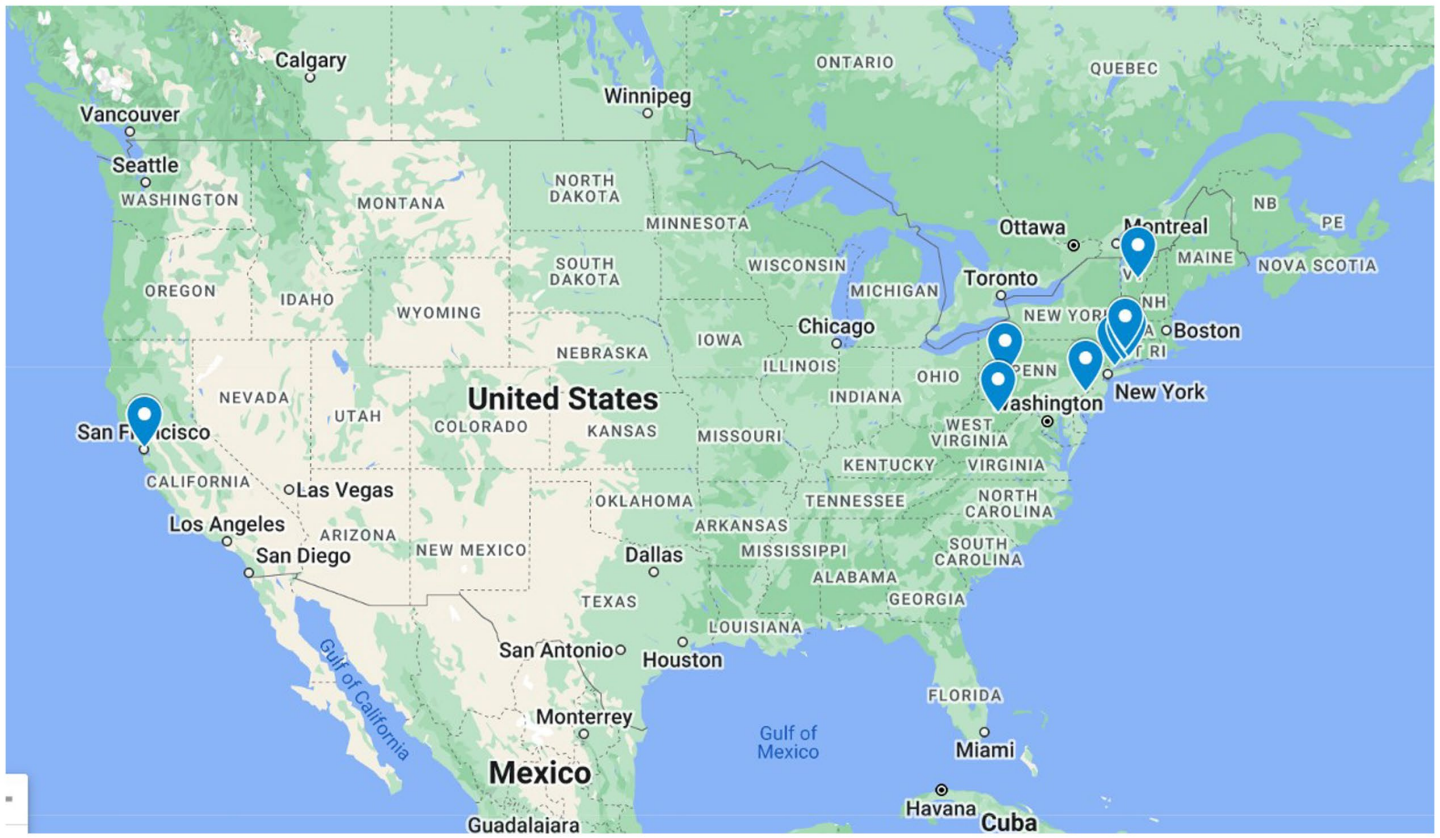
Distribution of places of publication of the newspapers in *ChroniclItaly 3.0* per title.

Despite these limitations, the collection arguably maintains an acceptable degree of balance between the representation of titles of different political orientation, geographical distribution, and numbers of issues throughout the period. Figure 2 shows the different places of publications of the newspapers collected in *ChroniclItaly 3.0.* The author nevertheless acknowledges that although the resource provides a reasonably comprehensive picture in the period of reference, discourse of migration produced by the Italian American community, issues such as over-or under-representation of some titles and potential polarization of topics may arise.

## Methodology

This study combines text mining techniques such as collocations and ngrams with Critical Discourse Analysis (CDA). Collocations and ngrams are used to identify relevant articles and passages in the corpus. Collocations are words that appear frequently within a certain distance of the search term and provide a picture of which words co-occur with other words in a corpus; ngrams are a contiguous sequence of two or more (n) items in a text. Ngram language models are probabilistic models –known as Markov chain (Gagniuc, 2017) –for predicting the next item in a sequence of elements in which the probability of each item depends on the proceeding one. These two methods complement each other; together, they provide a comprehensive overview of how words are distributed in the analyzed material and therefore they are especially helpful for exploring large quantities of unstructured textual data. Particularly for cases in which keywords searches are challenging, for instance because some concepts are difficult to define linguistically (such as return migration) (Oberbichler and Pfanzelter, 2022), these methods may reveal important insights into the concepts migrants associated with the notion of remigration as well as answer questions of discourse proliferation and awareness.

Guided by CDA theory, the narratives of remigration are analyzed in identified excerpts. Specific attention is given to how retuned migrants are discursively represented in the diasporic media and through which communicative strategies their identity is constructed. As in any crisis, crisis of identity are also always attempts at maintaining power (Viola, 2022). For example, when a host society feels threatened, concepts of “foreign” and “belonging” are used to justify specific ideologies and thus usually become explicit; but in contexts of return migration, individuals can paradoxically become strangers in their own country of origin. The analysis will therefore focus especially on understanding how processes of social inclusion and identity construction and representation unfold in diasporic contexts as well as within the same community. The first hypothesis is that contrary to the Italian national debate that simplistically framed emigration, return migration and remittances as the single solution to a problematic, social situation, the discourse of remigration in the immigrant press –though still mediated –was more ambivalent in that it was produced by the Italian diasporic community and therefore enacted as a consequence of its own migratory experience. The intention is to obtain a more intimate representation of the experience of return migration as a changing, sometimes traumatic experience, both for the returnees and the Italians who had never left. By analyzing the conflicting vectors of public discourse on migrants and discourse by migrants, the aim is to open up avenues for a critical reflection on emerging common as well as diverging themes and the impact these may have had on the general perception of return migration as a crisis of identity (second hypothesis).

## Analysis and results

This section shows the results of the analysis following the described methodology; in particular, the first section shows the results of the ngrams analysis while the second one analyzes identified excerpts using CDA.

### Ngrams

**Figure 3:**
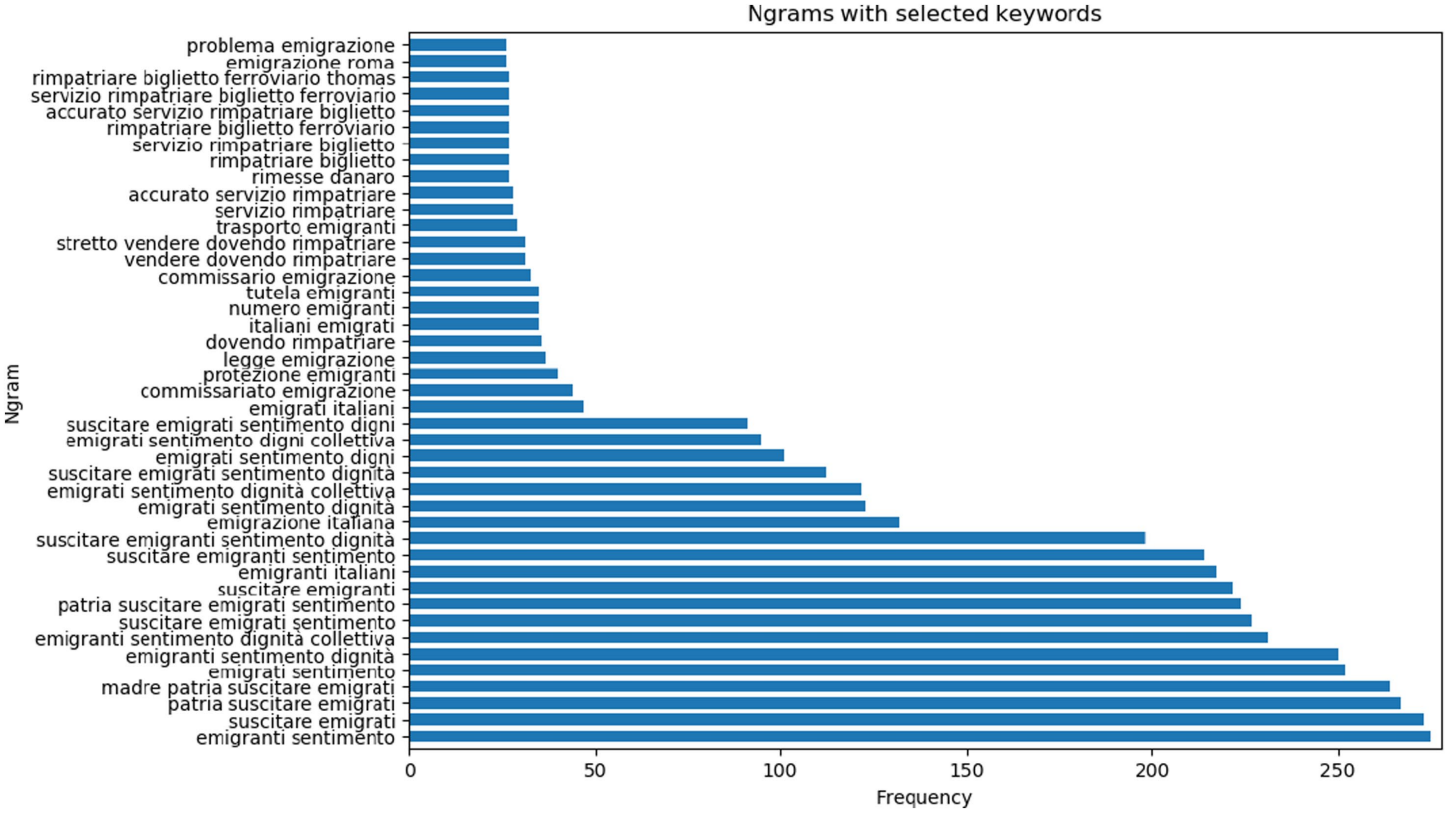
Frequency of distribution of N-grams in *ChroniclItaly 3.0* with selected words.

Ngrams (bi-grams, tri-grams and four-grams) are computed for salient words so as to gain insights on their semantic distributions in the corpus. The used words are *emigrazione* (emigration), *emigrant* (emigrants), *rimpatriare* (to repatriate), *ritornare* (to return), *rimesse* (remittances) in all possible morphological combinations (e.g., *ritornare*, *ritornati*, *ritornato*). The results are displayed in Figure 3.

A few interesting considerations can already be drawn from the results. First, ngrams containing the word ‘emigranti’ (emigrants) or ‘emigrati’ (emigrated) have the highest frequency of occurrence whereas ngrams in combination with ‘rimpatriare’ (to repatriate) occur much less frequently. This would suggest that the topic of emigration was discussed much more frequently than the topic of returning in turn suggesting that Italian migrants were more preoccupied with matters concerning their migratory experience rather than with returning to Italy. The finding indicates a contrast with the Italian public debate of migration which almost exclusively focused on the economic contribution migrants would provide to Italy upon return and on solving the southern question through remittances (here with the lowest rate of occurrence – ‘rimesse’).

**Figure 4:**
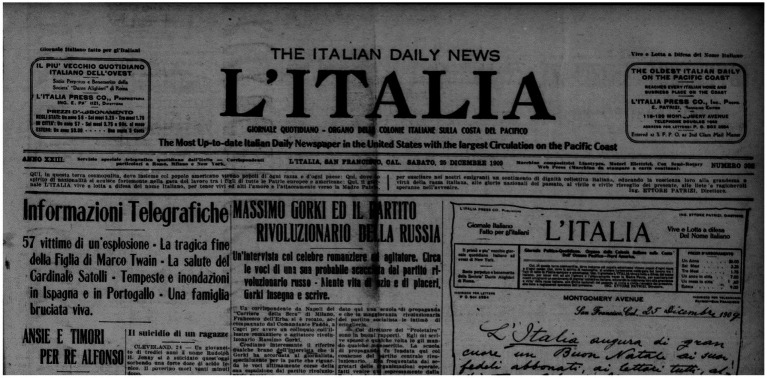
Frontpage of *L’Italia* 25 December 1909.

Second, ngrams containing ‘emigranti’ are almost always in combination with ‘sentimento’ (sentiment) and ‘patria’ (homeland). As it was custom at the time, ethnic newspapers’ front page often reported their mission statement; this would change periodically depending on the newspaper’s political orientation or indeed the historical moment. One of the newspapers in *ChroniclItaly 3.0* is *L’Italia*, a *prominenti* newspaper with the longest record of publication. Figure 4 shows the top part of *L’Italia* frontpage of 25 December 1909. The mission statement can be found right below the date. In the years 1909–1910, *L’Italia*’s mission statement was the following (emphasis mine):

*In questa terra cosmopolita, dove insieme col popolo americano vivono popoli di ogni razza e d’ogni paese; Qui, dove lo spirito di nazionalità si acuisce fortemente nella gara del lavoro tra i figli di tutte le Patrie europee e americane: Qui, il giornale L’ ITALIA vive e lotta a difesa del nome Italiano, per tener vivi ed alti l'amore e l’attaccamento verso la Madre Patria, per suscitare nel nostri **emigranti un sentimento** di dignità collettiva Italiana, educando la coscienza loro alla grandezza e virtù della razza Italiana, alle glorie nazionali del passato, al virile e civile risveglio del presente, alle liete e ragionevoli speranze nell’ avvenire.* ^10^

The mission statement pushes a narrative aiming at empowering the Italian migrant community by resorting to the cardinal concepts of an Italian heritage of civilization and a glorious past of grandeur. As it has been pointed out in the academic discussion (Cibotti, 1994; Deschamps, 2011; Vellon, 2017; Viola and Verheul, 2019a), such exaltation of the Italian nationalistic sentiment was not isolating the Italian diasporic community; rather, it was part of a wider strategy to construct an ideological concept of Italian identity that would create unity and exert political force to negotiate inclusion (Viola and Verheul, 2019b). Immigrant newspapers used such nationalistic strategies to unify one’s in-group and educate the Italian community accordingly. The mass migrations were profoundly impacting the socio-cultural landscape of the United States, most notably visible in the process of redefinition that was affecting social categories such as race, citizenship, and whiteness (Barrett and Roediger, 1997; Foley, 1997; Brodkin, 1998; Jacobson, 1998; Guglielmo and Salerno, 2003; Guglielmo, 2004). Italian immigrants were not spared from this process; they would often be victims of social discrimination, exploitation, physical violence, and even lynching (LaGumina, 1999, 2018; Connell and Gardaphé, 2010). The narrative found in *L’Italia*’s mission statement reflects this struggle to negotiate inclusion in the host society and it shows how this formed a substantial part of the Italian migratory experience.

Third, ngrams containing the word “rimpatriare” (to repatriate) score rather low in the corpus; again, this would suggest that the topic of resettling in Italy was not frequently discussed by the immigrant press. Even more interestingly, “rimpatriare” is found in combination with “dovendo” (being forced to), “servizio” (service), and “biglietto” (ticket). This could indicate that contrary to the national discourse of migration in Italy for which migrants were expected to return, in the immigrant press repatriation was framed as a forced, rather than a voluntary decision to return. It would also indicate that immigrant newspapers were mostly discussing remigration in the context of giving the Italian community practical information about returning to Italy. The CDA of selected excerpts in the next section will offer a finer grained picture of the different narratives in the repository.

### Critical discourse analysis

Collocations lists are generated from the same search terms used to identify salient ngrams and relevant excerpts are retrieved from these lists. CDA is then applied to analyze these linguistic data as discursive “events” (Reisigl and Wodak, 2001), that is as realities in which the social, political, and historical context in which they are embedded is accounted as co-producer of the narratives. The aim is to obtain a richer, more nuanced and intimate perspective of the experience of migration and remigration from the point of view of the migrants as it was shaped by the social context in which the diasporic communities were entrenched. Excerpt 1 is taken from *L’Italia* on 18 August 1904 (emphasis mine).

1. *È da augurarsi che la burocrazia degli uffici ministeriali **tanto lenta quanto è imbecille**, non frapponga inciampi e ostacoli all’esecuzione di disegni e di proposte, mercè cui la **difesa della emigrazione** e la sua tutela **contro le male arti delle arpie** nazionali e straniere esca dal regno della retorica, e diventi realtà immanente ed efficace. […] Non dimentichino in Italia che la popolazione Italiana del Nord America si accosta al milione e che, specialmente nel Mezzogiorno della Penisola, se interi paesi pagano le tasse o non muoiono di fame é **in virtù delle rimesse degli emigranti**!*
2. Let us all hope that the bureaucracy of the ministerial offices, **as slow as it is idiotic**, will not create hurdles and obstacles interfering with bills and proposals, and that the **defense of emigration** and its protection against the **evils of national and foreign sharks** will leave the kingdom of rhetoric to soon become an efficient reality. […] May Italy not forget that the Italians in Northern America are about one million and that, especially in the *Mezzoggiorno*, if entire towns are able to pay the taxes or not starve is **by virtue of the emigrants’ remittances**!

More than 3 years after the law on emigration was passed, migrants’ conditions had not improved much. Excerpt 1 shows a feeling of frustration, resentment, and powerlessness by the Italian American community towards the Italian Government about the lack of progress towards effective reforms. One of the main novelties introduced by the law had been the centralization of all matters regarding emigration through the establishment of the *Commissariato Generale per l’Emigrazione*. The passage above refers precisely to a visit to the United States by Adolfo Rossi, Inspector of the general commissariat for emigration at the time. The purpose of the visit was to assess the conditions of the Italian colonies, especially outside of the biggest cities, to explore the possibility of establishing employment offices that would give Italian migrants accurate job information and protect their interests on their behalf. Indeed, like millions other migrants, Italians were often exploited as cheap labor and forced to work in extremely poor conditions. The argument in favor of the law had claimed that the reform would have assisted migrants in all stages of their migratory experience, including providing support in the host countries. But in reality, changes had been slow to implement. The passage highlights how Italian Americans were disillusioned by the moderate impact of these reforms and how they were feeling neglected and essentially misunderstood.

The reference to remittances is also significant; it shows that the Italian diasporic community felt exploited by the Italian Government. Emigrants thought that they were significantly contributing to the Italian national economy by sending remittances to their families in Italy. Yet despite their important contribution –perhaps exaggerated in the article –their efforts were not appreciated by the Government which was lost in rhetoric and slow bureaucracy.

Excerpt 2 is taken from an issue published by *L’Italia* 2 years after, 14 November 1906 (emphasis mine).

1. *L’intensità e l’eccesso del lavoro imposti dalla **povertà** che esiste fra i nostri connazionali in ragione **diretta delle loro rimesse postali** e che da queste viene tragicamente mascherata.*
2. The intensity and excess of work imposed by **poverty** on our fellow Italians is **directly due to their remittances** and by them is tragically concealed.

The excerpt above provides a sad account of the harsh living conditions of Italian Americans in the period of mass migrations; it also gives a more bitter view on remittances than the exalted narrative the national discourse was spreading. The passage shatters the illusion that migrants were becoming rich and that they were supposedly able to save large amounts of money easily and without sacrifices. Migrants knew that this fabricated belief tragically concealed a much darker reality. The pressure of saving as much money as possible to send remittances to Italy forced them to work excessively and to live in extreme poverty. The sharp contrast with the national discourse suggests that Italian migrants felt their struggles were minimized or even denied and that their experiences were misunderstood.

The following excerpt taken from *La Tribuna del Connecticut* of 27 April 1907 describes the constant tension of the migrant’s condition, the longing for a beloved, yet resented country that negates them everything and forces them to leave. This ambivalence remains sustained even when, crushed by nostalgia, the migrant decides to return (emphasis mine).

1. *Poverino!. **Tutto gli ha negato la patria**! Il lavoro, il pane, il tugurio, il vestito! Ed egli segue la corrente, che fuori della patria trascina tante preziose esistenze, che **toglie alla patria i migliori dei suoi figli**! Parte per raggiungere l’ignoto, pieno di fiducia, di speranza, perche’ ha inteso che la’, lontano, lontano, oltre l’oceano, c’e’ una terra dove **l’oro si guadagna a palate**. E parte … Mentre il piroscafo solca le onde, […] guarda, e protende minaccioso il pugno verso **l’ingrata** che gli ha negato il pane! Il povero emigrante si volta ancora, non vede più’ quel punto, che […] adesso gli e’ caro; e il ricordo […] gli da’ il capogiro, il cuore palpita, le lagrime gli fanno velo agli occhi. […] E guardando sempre scorge finalmente la terra, e’ l**’America**! L’America, la meta agognata, la **terra che gli deve procurare quello che la patria gli ha negato**. Arriva, sbarca, lavora, accumula capitali, spedisce denaro ai suoi, ha il benessere, ha tutto. Ma… un punto oscuro e’ sempre nella sua mente, il suo cuore ha un palpito perenne’. Egli dovrebbe esser contento e’ non lo e’! […] **E’ il ricordo della terra natia**. […] Ma che m’importa della terra natia, egli dice […] E’ la lontananza dei miei cari. Se li avessi vicino a me ritornerebbe la calma. E spinto da una repentina risoluzione s’imbarca e inaspettato tocca la terra natia. Rivede i suoi cari, e al suo cuore ritorna la calma. **Ma la patria e’ sempre quell’ingrata**, **ed egli insieme ai suoi ritorna in America**. Ora li ha tutti vicini a se, ora é tranquillo. Ma egli **s’inganna**, egli mente a se’ stesso, sente sempre dentro di se qualcosa che lo conturba. La malinconia l’invade sempre. **Lo perseguita il ricordo della terra natia** […] Non può’ più mentire a sé stesso. **E’ la patria lontana, è la nostalgia**.*
2. Poor thing! **His homeland denied him everything**! Work, bread, hovel, clothes! And he follows the current, which carries so many precious lives out of the homeland, which **takes away its best children**! He leaves to reach the unknown, full of trust, of hope, because he has understood that over there, far, far away, beyond the ocean, there is a land **where gold can be earned by the shovelful**. And he leaves … While the steamer cuts through the waves, every now and then he looks back […] and holds out his fist threateningly towards the **ungrateful land** who denied him bread! […] The poor emigrant turns around again, he no longer sees that point, which […] is now dear to him; and the memory […] makes him dizzy, his heart flutters, the tears veil his eyes. […] As he keeps looking, he finally sees the land, it’s **America**! America, the craved land, **the land that will give him what his homeland denied him**. He arrives, he disembarks, he works, he accumulates capital, he sends money to his family, he lives well, he has everything. But a dark thought is always on his mind, his heart flutters perpetually. He should be happy and he’s not! […] It is the memory of the native land. […] But what do I care about my native land, he says […] It’s the distance from my loved ones. If I had them close to me, I’d be at peace. And driven by a sudden resolution he embarks and unexpectedly reaches his native land. He sees his loved ones again, and calm returns to his heart. But **the homeland is always ungrateful**, and **he and his family return to America**. Now he has them all close to him, now he is calm. But **he deceives himself**, he lies to himself, he always feels something inside that disturbs him. Melancholy always invades him. The memory of his native land haunts him […] He can no longer lie to himself. **It’s the distant homeland**, it’s nostalgia.

The passage above well exemplifies the tormented condition of the migrant, the perennial crisis of identity that does not allow them to fully enjoy even a wealthy life. The migrant adores the native country but at the same time, they resent it, they feel rejected by it, their country “denied them everything.” As they leave, they are overwhelmed by these two conflicting feelings. This tension is also conveyed linguistically in the text. Whereas America is expressly mentioned, Italy is always referred to as “the homeland.” This not only highlights a more intimate relationship with the home country, but also a clear opposition with the host country. This narrative thread is sustained throughout the entire passage: the homeland is “ungrateful,” America is ‘craved’; the homeland denies the migrant everything, America is the promise of gold. When the migrant returns, they sadly discover that the homeland has not changed at all, it has remained “ungrateful.” They must leave again, they must **return**, but this time to America.

This passage illustrates the second hypothesis of this study, that migration experience did not end with repatriation. The migrant described in the excerpt may well be the migrant of investment (Cerase, 1974), the one who has done well abroad and could innovatively contribute to the society of origin when returning to it. But upon return, the migrant experiences an old sense of crisis; just like before the departure, the homeland does not welcome the migrant’s contributions, nor is it grateful for the sacrifices the migrant has made. For this migrant to leave is, once again, the only alternative. This time, however, the migrant takes the family with them, marking the process as definitive. This more nuanced experience of migration narrates a complicated negotiation of identity between the migrant and the homeland within the wider experience of migration. The migrant will always long for their homeland but something has changed. The migrant understands that their torment is not a desire to go back, it is nostalgia.

Excerpt 4 is taken from *L’Italia* of 16 November 1908 (emphasis mine).

1. *L’ **italiano ritornato** in patria, di regola è praticamente male avvezzato. Egli ha **perduto le sue buone qualità italiane**, guadagnando le cattive americane, conservando sempre le peggiori, e non le migliori qualità dei due paesi.*
2. **Italians who return** home have normally bad habits. They have **lost their good Italian qualities** and learned the bad American ones, always keeping the worst, and not the best qualities of both countries.

The excerpt describes repatriated Italians as having been worsened by migration, especially morally. The narrative is built along the ‘Us vs. Them’ paradigm synthesized in the culturalized images of country-specific qualities, which are positive if referred to the in-group and negative when referred to the Other. This discourse path highlights the differences between the two groups, rejects the values of the Other, and builds confidence in the “us” group. But the passage shows another interesting phenomenon: in the context of return migration, the returnees become themselves “the Other.” Because they have lost what indeed made them Italian (the good Italian qualities) in favor of ‘bad’ American habits, now their right to belong is contested. The “us” vs. “them” narrative emphasizes the in-group identity which in fact refers to the Italians who never left. The process of transformation migrants have gone through is oversimplified and reduced to a linear, binary opposition: Italian = good; American = bad. This description of how returnees were perceived in Italy suggests that their process of social inclusion was complex even within their community of origin. Within this discourse frame, Italians who left were no longer considered Italian, suggesting that once returned, migrants struggled again with their identity negotiation.

Excerpt 5 is taken from *L’Italia* of 19 July 1912 (emphasis mine).

1. *Facendosi l’esame di coscienza, l’Italia deve confessare di **non aver ancora pagato iI proprio tributo all’emigrazione**. Quando molti si domandano se l’emigrazione sia un bene o un male, si puo’ rispondere paradossalmente — ma non meno veracemente — che l’emigrazione è un bene per la patria, ma **un male per i suoi figli**. È l’individuo che si sacrifica per la collettività. Le **vittime dell’emigrazione** in tempo di pace sono assai piu’ numerose delle vittime della guerra.*
2. If Italy examined its own conscience it should confess to **not having paid its dues to emigration** yet. When many ask if emigration is good or bad, one can answer paradoxically-but not less sincerely –that **emigration** is good for the country, but **bad for its children**. It is the individuals that sacrifice themselves for the sake of the collectivity. The **victims of emigration** in peacetime far outnumber war victims.

The article specifically refers to the victims of tuberculosis which at the time was affecting the migrant communities. According to the ship’s logs of ships from America to Italy, about 50% of all Italian passengers were affected by tuberculosis (Padovani, 1909). This was because once they would fall ill, migrants could not work anymore, and because typically they could not afford medicines, they would either voluntarily try to repatriate to be cured by their families or they would be expelled by the host country. One out of three of these migrants would die during the journey (*ibid.*). Those who would survive, however, would become a liability both for national and local governments as they represented an economic burden and a threat to national health security. Their families also considered them as a failure as they had become reason for embarrassment in the community (Molinari, 2017). The excerpt presents a similar narrative of examples 1 and 2 analyzed above: migrants frame themselves as victims. Using the war metaphor, migrants are in fact war heroes who sacrifice themselves for their country. The article indicates acceptance of emigration as a necessary evil: Italy is a mother who must lose her children to survive. This feeling of acceptance is however far from being uncritical; the passage indeed reveals an element of resentment towards Italy for not recognizing the enormous sacrifices Italian migrants were enduring for the greater good. Again, the conveyed message is that of conflicting worldviews in which migrants felt exploited and misunderstood.

Excerpt 6 is taken from *La Sentinella* of 26 October 1929 (emphasis mine).

1. *Un carattere precipuo distingue gl’italiani immigrati in America dagl’italiani emigrati altrove. All’inizio erano tutti accomunati da una sola speranza: **fare o rifare le proprie finanze e rimpatriare**. […] Fra gli italiani d’America **il fine del rimpatrio** è, salvo le eccezioni, **cessato**, per necessità di cose, per logica e diritto di famiglia. […] Qui non vi sono più, e non vi possono essere “italiani d’America, “ma v’è un Gruppo italo-americano, che ha trovato inevitabile costituirsi parte integrante della popolazione “at large,” composta di altri sessantasette gruppi di razza. La nostra **italianità**, quindi, è divenuta un sentimento, un **fenomeno, più ampio**. **Noi praticamente non siamo più italiani** da un punto di vista nazionale, ma dal punto di vista della razza, che comprende la nazionalità. I nostri figli, nati in America, sono già più numerosi di noi, e poiché, come dice il Diritto Romano, che, in fondo, sanziona un sentimento naturale, “amor descendit, “**nazionalmente noi ci sentiamo attaccati più alla terra dei nostri figli che a quella dei nostri padri**.*
2. A specific character distinguishes Italians who immigrated to America from Italians who emigrated elsewhere. At the beginning they all had one hope in common: **to raise their finances and repatriate**. […] Among Italian Americans the **purpose of repatriating has**, with a few exceptions, **ceased**, because of necessity, logic and family law. […] Here there are no longer, and there can no longer be “Italians from America, “but there is an Italian-American group, which found it inevitable to form an integral part of the population “at large,” made up of sixty-seven other groups of race. Our **Italianness**, therefore, has become a **broader feeling**, a phenomenon. **We practically are no longer Italians** from a national point of view, but from the point of view of race, which includes nationality. Our children, born in America, are already more numerous than us, and since, as Roman Law says “amor descendit, “after all sanctioning a natural feeling, nationally we feel more attached to the land of our children than to that of our fathers.

The excerpt illustrates the identity negotiation struggle of Italian migrants. After a few years in America, migrants’ sense of identity has been changed by the migratory experience. They are no longer ‘Italians who live in America’; the sentence highlights the opposition between Italy intended as manifestation of a cultural identity (‘Italians’) and America, intended as a place. The process of transformation has deeply affected their Italianness which has transformed into something bigger. Now migrants’ identity incorporates American identity too (‘there is an Italian-American group’) and this allows them to negotiate inclusion within the wider American society (‘integral part of the population at large’). It is interesting to notice how the process of identity negotiation also affected migrants’ decision to repatriate. Initially they all wanted to return to Italy, but now this path is chosen only by a few exceptions. This new identity is also passed to the new generations, thus strengthening a ‘natural feeling’. This discourse frame abandons the “us” vs. “them” narrative of the previous years. In this passage, there is no opposition between the in-group and the out-group identity; the complex process of social inclusion is mediated by a newly formed identity which is no longer in crisis.

## Discussion

The combination of text mining and CDA allowed for the identification of recurring themes and characteristics in how the experience of migration and remigration was perceived by Italian American migrants. The ngrams analysis of the semantic distribution of salient words such as *emigrazione*, *emigranti*, *rimpatriare*, *ritornare*, *rimesse* suggested that in the ethnic press, repatriation was less frequently discussed than matters of emigration and life in America. Ngrams containing the word ‘remittances’ scored low in the corpus suggesting a contrasting trend with the Italian public debate of migration in which remittances were at the centre. Moreover, these are found in combination with words such as ‘service’ or ‘ticket’ which could indicate an emphasis on practical information and advice in relation to repatriation. This would suggest a stronger preoccupation with the enormous social challenges migrants had to face in America rather than with repatriating. Migrants often struggled to navigate the complexities of a constantly evolving migration landscape, both in Italy (e.g., the law of 1901) and the United States. The finding would therefore also be in line with the historical mission of the ethnic press: primarily to help Italians cope with life in the host country whilst maintaining a bond with their heritage.

The CDA allowed for the triangulation of linguistic data within their social contexts and concurrent socio-historical events. The analysis of excerpts provided deeper insights into how the Italian diasporic community in the United States was narrating its own migratory experience. Specifically, it showed a common pattern of a sense of crisis and identity negotiation manifested through feelings of being misunderstood, rejected and unappreciated and constructed around three main themes: 1) the Italian Government was profiting from emigration; this narrative was found in reference to remittances; 2) Italian migrants were heroes who were sacrificing themselves for the greater good; this narrative was found in reference to the struggles in the host country, including poverty and tuberculosis, and 3) Italian migrants had changed; this narrative was found in reference to returnees or returning to Italy. Generally, the adopted discourse strategy was constructed around an “us” vs. “them” narrative, i.e., Italian migrants vs. the Italian Government but also Italians vs. Americans (particularly in early years) and Italian migrants vs. Italians. The criticism of Italy and the Italian Government as Other-identity was found to be a common communicative device used by Italian migrants to position themselves as the ‘sacrificial victims’, the heroes who were singlehandedly rescuing the country. Therefore, if in the national debate migrants and emigration were framed as beneficial for the country and migration was oversimplified as an easy way to make money, in the ethnic press the same frame is used to criticize such praises as plainly rhetorical, to characterize migration as an extremely painful process for the individual, and the Italian Government as being unable to factually demonstrate appreciation for the migrants’ sacrifice.

The analysis also highlighted how migrants were struggling with processes of inclusion and identity when returning to Italy. Returnees were framed as having lost their ‘italianness’ and as having been worsened by the migratory experience. They were described as hybrid creatures made up of the worse traits of the two countries. The derogatory image polarizes differences between Italian and American values first and between the so-transformed Italians and Italians after. This narrative is once again in contrast with the national discourse for which repatriated migrants were believed to being improved by the migratory experience and in turn praised for improving Italy. Migrants also described themselves as feeling rejected by the society of origin when returning. The homeland was characterized as ungrateful, again not appreciative of the migrants’ sacrifices and feelings. Returnees were on the contrary represented as ‘foreign’, suggesting that upon return, migrants faced new, great challenges and that these individuals may have experienced remigration as a second crisis of identity. It also supports the second hypothesis of this study, that for many individuals remigration was a bi-directional movement which often did not stop with repatriation.

Processes of identity negotiation and inclusion were found to be a constant preoccupation of the migratory experience. References to Italian and American identity, Italianness, and Italian American identity indicate a continuous search for acceptance and suggest a less material reading of the reasons behind migration, that is beyond economic reasons. Ethnic newspapers revealed that from a cognitive point of view, what made the displacement process particularly painful for the migrant –even when they could enjoy good social and living conditions –was a constant sense of identity crisis, a feeling of rejection, be it from their homeland or the host society. It is only when the migrant made peace with this feeling, only when they finally embraced a new identity that they were no longer in crisis and migration –intended as a painful process –was finally concluded.

## Conclusion

At the turn of the twentieth century, the national debate of migration in Italy became entangled with the so-called Southern Question, that is the economic integration of the South. The predominant –though not exclusive –view was that mass emigration was fatalistically necessary to relieve the South from unemployment; moreover, thanks to remittances, emigration was beneficial for the country, as well as remigration, since returning migrants were coming back with money and skills. The exaggerated value of remittances became at the centre of the discourse, almost creating the myth that remittances alone would solve all Italy’s problems. This article offered a novel perspective of the migratory experience of Italian migrants by using for the first time ethnic newspapers to compare migrants’ discourse of emigration, remigration, and remittances against the national debate of emigration and remigration in Italy. This insider’s perspective showed that while the Italian public discourse praised and actively encouraged remigration as positive for the country, both economically and socially, Italian Americans’ views were more complex and ambivalent. This more inner perspective –though still mediated –unveiled the identity construction mechanisms part of those strategies which, historically, were implemented by the Italian diasporic community to construct identity, negotiate inclusion, and maintain power in a hostile environment, may this have been the host or the country of origin. In this way, the study provided a more nuanced and discursive conceptualization of return migration as a bidirectional phenomenon deeply entrenched in identity negotiation processes.

The study also offered a methodological contribution to digital migration studies. A mixed-method approach of distant (ngrams and collocations) and close reading (CDA) was used to explore how migrants themselves were experiencing migration, how their positioning was constructed in relation to the homeland, and how they coped with identity negotiation struggles. Text mining and semantic modeling methods facilitated a more immediate identification of relevant passages, whereas the semantic similarity clustering (ngrams) allowed for a general overview of the distribution of salient words in the corpus. Finally, CDA allowed for a finer-grained analysis of the narratives.

The findings of this study provided a comprehensive understanding of the dynamics, motivations, and challenges associated with migrants returning to their home countries. This knowledge may contribute to effective migration management and policy development, to ensure the well-being of both migrants and the societies they return to, making it highly relevant in the EU and the broader context of global migration. In this respect, the study demonstrated the value of the ethnic press as a novel source that can add an intimate dimension to the study of migration of the past century, of the migratory experiences of those migrant communities and their process of identity negotiation. It highlighted the considerable discrepancy between the exalted domestic discourse about remigration and the more nuanced experience of Italian migrants. Such discrepancy, the article argued, may be seen as an indication that the Italian Government’s view of remigration –mainly through the lens of domestic economic advantage –deeply underestimated the complexity of migration as a social phenomenon and as a profoundly changing psychological experience, also for the Italians who never migrated. In the long run, this error of judgment deeply damaged Italy as many of those *ritornati* felt misunderstood, rejected, and disillusioned and crossed the Atlantic again, this time never to return.

## Data availability statement

Publicly available datasets were analyzed in this study. This data can be found at: https://zenodo.org/record/4596345.

## Author contributions

The author confirms being the sole contributor of this work and has approved it for publication.

## Footnotes

^1^The U.S. Congressional commission responsible for investigating immigration.

^2^The term banchisti refers to a specific group of Italian Americans who claimed to be bankers but profited from the situation of uneducated and naive Italian migrants. These individuals may have exploited their fellow immigrants through deceptive financial practices or scams. In many cases, these individuals took advantage of vulnerable newcomers who were unfamiliar with the financial system and its regulations in their new country. This type of exploitation is not unique to Italian immigrants but has been observed in various immigrant communities.

^3^Although these two categories include the majority of the Italian American newspapers of the period, there were also some self-claimed politically independent publications such as *The Patriot*, *La Sentinella del West* and *The Independent. ChroniclItaly 3.0* includes them all.

^4^For a more in-depth discussion of these two types of publications, see Vellon (2017), Viola and Verheul (2019a,b), Deschamps (2020), Viola (2021).

^5^ https://zenodo.org/record/4596345#.ZBCEcXbMI2w

^6^ https://chroniclingamerica.loc.gov/lccn/sn84020351/

^7^ http://www.loc.gov/chroniclingamerica/lccn/sn92051386/issues

^8^ https://chroniclingamerica.loc.gov/lccn/sn93053873/holdings/

^9^ https://chroniclingamerica.loc.gov/

^10^“In this cosmopolitan land, where people of any race and from any country live together with the American people; here, where among the sons of all European and American countries the spirit of nationality becomes stronger in the hunt for a job: here, the newspaper L’ITALIA lives and fights to defend the Italian name, to keep the love for and attachment to the Homeland high and alive, to inspire in our **emigrants a sentiment** of Italian collective dignity, educating their consciousness in the greatness and virtue of the Italian race, in the glories of the past, in the virile and civil awakening of the present, in the good and reasonable hopes for the future.” In this article, unless otherwise stated all translations are by the author.
